# Learning joints relation graphs for video action recognition

**DOI:** 10.3389/fnbot.2022.918434

**Published:** 2022-10-11

**Authors:** Xiaodong Liu, Huating Xu, Miao Wang

**Affiliations:** School of Software, Henan Institute of Engineering, Zhengzhou, China

**Keywords:** action recognition, deep learning, convolutional neural network, GCN, joints relation

## Abstract

Previous video action recognition mainly focuses on extracting spatial and temporal features from videos or capturing physical dependencies among joints. The relation between joints is often ignored. Modeling the relation between joints is important for action recognition. Aiming at learning discriminative relation between joints, this paper proposes a joint spatial-temporal reasoning (JSTR) framework to recognize action from videos. For the spatial representation, a joints spatial relation graph is built to capture position relations between joints. For the temporal representation, temporal information of body joints is modeled by the intra-joint temporal relation graph. The spatial reasoning feature and the temporal reasoning feature are fused to recognize action from videos. The effectiveness of our method is demonstrated in three real-world video action recognition datasets. The experiment results display good performance across all of these datasets.

## Introduction

With the rapid development of internet and mobile intelligent devices, multimedia applications, including urban security, medical treatment, education, communication, industrial production, and cultural film, are more and more widely used. They generate huge amounts of videos every hour and now account for about 80% of the data. YouTube has one billion users, with 300 h of video uploaded and 50 million h of video watched per minute. In addition to Internet video, surveillance video is also an important source of video data. There are about 30 million surveillance cameras in China, and 60 EB of video data are generated per month. Thus, how to effectively analyze and understand these data and create greater economic and social benefits has been a hot topic.

Video action recognition is one of the most important tasks for comprehensively understanding video content. It has many potential applications, such as human–robot interaction (de Carvalho et al., 2022), fruit picking (Wu et al., 2022), agricultural (Tang et al., 2020), and warning decision (Fang et al., 2021). There are various attempts at video action recognition based on spatial and temporal features. Two-stream convolutional neural network (CNN) (Simonyan and Zisserman, 2014) consists of spatial and temporal sub-networks. The spatial streaming sub-network extracts a static feature of the video frame, and the temporal streaming sub-network extracts optical flow features containing motion features of the video. Then, the spatial and temporal features are aggregated, and the action feature of the video is produced. Although the two-stream CNN can utilize the spatial and temporal features of the video, there are some limitations. First, spatial CNN only extracts a single video frame and the information of the rest video frames is not utilized. Second, optical flow extraction is very time-consuming. To avoid the waste of video frames, Wang et al. (2017) fuse spatial features of all video frames and optical flow features to improve the accuracy of action recognition, and this method also needs to consume too much time. 3D Convolutional network (C3D) (Tran et al., 2015) expands the two-dimensional convolution neural network to three-dimensional, which can learn both spatial and temporal features. Its performance is better than two-stream CNN and does not need to extract optical flow. However, C3D adds many weight parameters and needs more training data. P-CNN (Chéron et al., 2015) clips the body joints in the video frame based on human pose estimation and extracts the spatial and temporal features of each body joint by CNN. This method trains an independent CNN network for each joint and fuses the temporal and spatial features extracted from different joints. Although this method successfully captures local information, the internal dependencies between joints are not modeled. To capture the internal dependencies between joints, recent methods (Li M. et al., 2019) construct a skeleton graph and apply graph convolution networks (GCN) (Defferrard et al., 2016) to extract correlated features. However, the internal relationship between the evolution of high-level semantics has not been established, and lacks the middle layer for modeling the semantics of the target joints, so the potential of using joints of the semantics in video action recognition based on the deep learning method cannot be fully exploited. Although the research on video action recognition has got a lot of achievements, it still faces some challenges.

**Figure 1:**
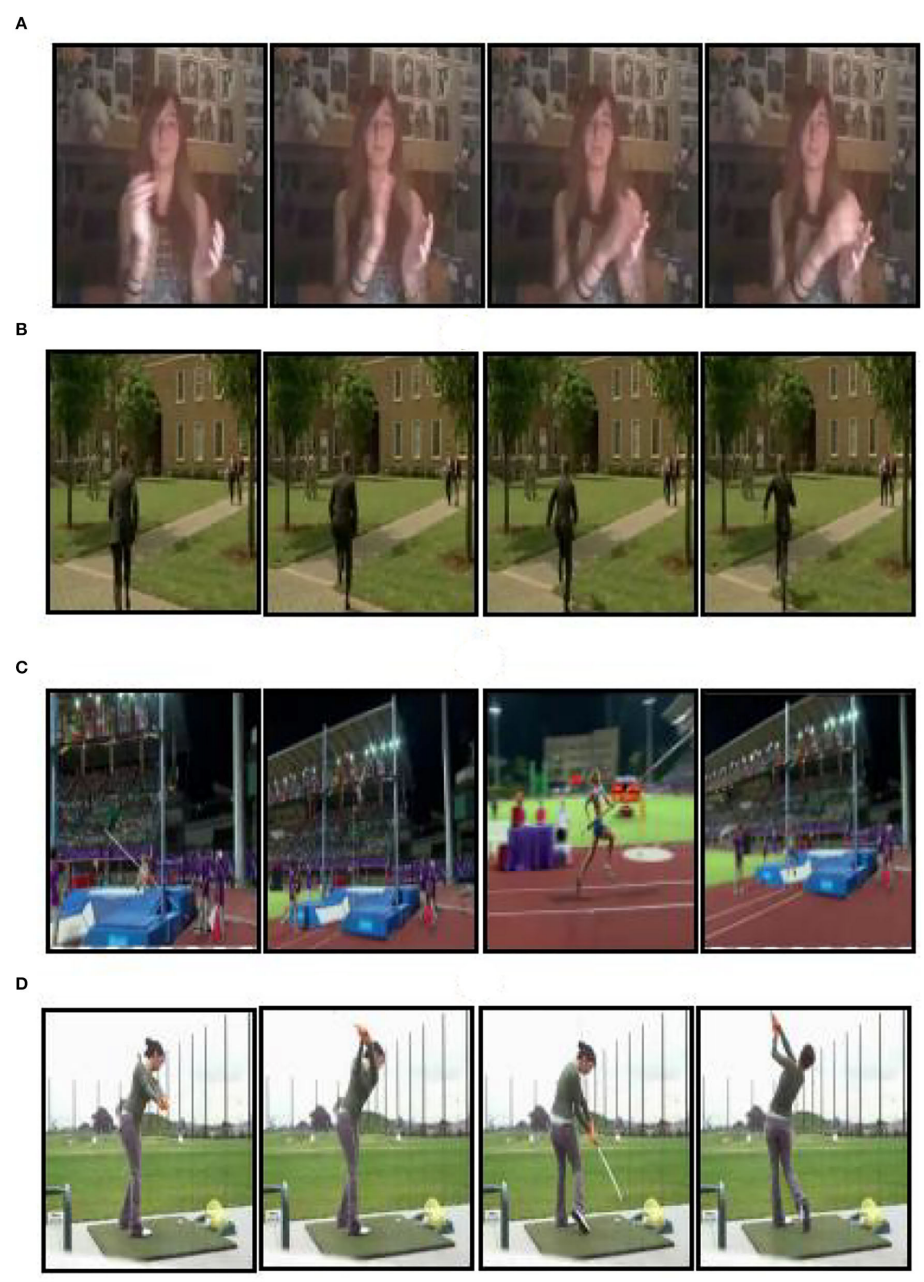
**(A–D)** The motivation of JSTR.

First, the action of the video is mainly depending on the movement of body joints. As an example, let us try to estimate the action of these videos in Figure 1. In Figure 1A, we mainly judge the action of the video by the movement of the hand and elbow. Other information can as an effective supplement to video action recognition. Similarly, detailed estimations can be made in Figures 1B–D. Therefore, how to make full use of the movement of the body joints is a significant challenge for video action recognition.

Second, the movement of body joints is the low-level feature. It contains less information than high-level semantics. If we can extract the movement of high-level semantic of body joints, the action recognition accuracy can be further improved. Therefore, how to model the movement of high-level semantics of body joints is another challenge for video action recognition.

To solve the problem of modeling the semantics of body joints and their movement evolution, this paper proposes a joint spatial-temporal reasoning (JSTR) framework for video action recognition. The JSTR consists of two sub-network. One is the pose estimation sub-network, which is used to generate the position of body joints. The other is the action recognition sub-network which takes as input the results of the pose estimation sub-network, and features of body joints are generated. Then a joints spatial reasoning (JSR) graph and intra-joint temporal relation (IJTR) graph are built, respectively, which is used to capture position and temporal relations between joints. Finally, the spatial reasoning feature and the temporal reasoning feature are fused to recognize the action from videos.

In summary, the contributions of this paper include:

- A geometric constraints-based human pose estimation method is proposed to improve the accuracy of human pose estimation.
- We design a joints spatial reasoning network for capturing position relation between body joints.
- We design an intra-joint temporal relation network for capturing the temporal evolution of body joints.

To verify the effectiveness of our proposed method, we compare the performance with the state-of-the-art method on three real-world video action recognition datasets: UCF101, HMDB51, and Kinetics. In the experiments of UCF101 and HMDB51, we obtain an excellent accuracy of 96.3 and 72.4%, respectively. In particular, on the large-scale kinetics dataset, we obtain an excellent top-1 accuracy of 75.8% and the top-5 accuracy of 92.6% on the validation set.

The rest of this paper is organized as follows: Section Related work presents the related work. Section The proposed methods gives a detailed description of the proposed method. Section Experiments shows the experimental results. Finally, Section Conclusion summarizes our conclusions and gives future work.

## Related work

There are various attempts at video action recognition based on spatial and temporal feature fusion. Feichtenhofer et al. (2016a,b) fused CNNs both spatially and temporally and then combined them with ResNets (He et al., 2016) to extract better spatio-temporal features. Sun et al. (2015) propose factorized spatio-temporal convolutional networks that factorize the original 3D convolution kernel learning as a sequential process of learning 2D spatial kernels. The computation cost of 2D CNN is low, but it cannot model the time information, so the accuracy is relatively low. 3D CNN can model time information and can obtain better accuracy, but the computation cost is too high. TSM (Lin et al., 2019) can facilitate information exchanged among neighboring frames by shifting part of the channels along the temporal dimension. It can be inserted into 2D CNNs to achieve temporal modeling, which balances accuracy and computation cost. STM (Jiang et al., 2019) contains a channel-wise spatial-temporal module to present the spatial-temporal features and a channel-wise motion module to efficiently encode motion features. TEA (Li et al., 2020) was designed to capture both short- and long-range temporal evolution by a temporal excitation and aggregation block. However, different features have different contributions to video action recognition. The difference in the contribution of different features on video action recognition cannot be fully utilized by simply combining the different features.

Attention mechanism can make full use of the difference in the contribution of different features. Simonyan and Zisserman (2014) uses both RGB and stacked optical flow as appearance and motion information, respectively. The accuracy is significantly boosted by simply fusing probability scores. VideoLSTM (Li et al., 2018) is an end-to-end sequence learning model, in which hard-wires convolution in the soft-attention LSTM. Xiang et al. (2018) propose a local feature integration framework based on a purely attention-based local feature integration, and carefully analyze the effect of different attention mechanisms. The attention mechanism can focus on important features and meanwhile ignore irrelevant signals and noise, so that the recognition accuracy can be improved.

The internal dependencies between human body joints are often ignored by the traditional action recognition methods, which will result in missing abundant information. To capture dependencies between joints, recent methods apply graph convolution networks (GCN) to extract correlated features. ST-GCN (Yan et al., 2018) learn spatial-temporal features simultaneously. ST-GCN mainly tries to aggregate wider-range features, but node features during long diffusion might be weakened. AS-GCN (Li M. et al., 2019) attempts to capture richer dependencies among body joints and useful non-local information. MUSLE (Li D. et al., 2019) builds space-time graphs and clusters the graphs into compact sub-graphs on each scale. In this paper, the JSTR model position and temporal relations between joints.

## The proposed methods

### Overview

**Figure 2:**
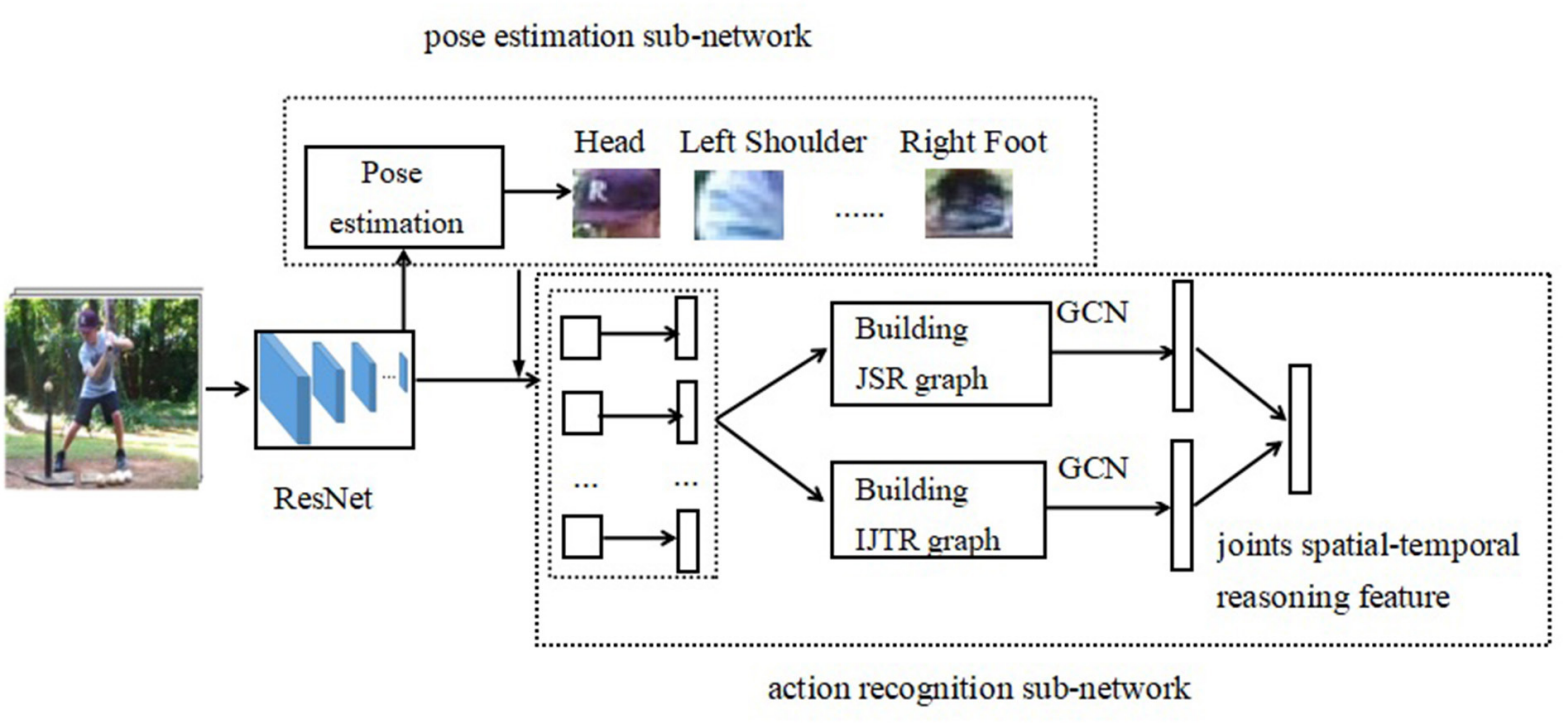
The framework of JSTR. The video frames are fed into a backbone network, such as Resnet. The last convolution layer feeds into a pose estimation sub-network and generates the position of body joints. The features of each joint are extracted by RoIAlign according to the positions of body joints. Upon these original features and positions of joints, a spatial-temporal reasoning graph is built. Then, the spatial features and temporal features are fused to recognize the action from videos.

The overall network framework of the joints spatial-temporal reasoning framework (JSTR) is illustrated in Figure 2. It consists of the pose estimation sub-network and the action recognition sub-network. First, we uniformly sample a set of T frames from the video and extract their feature vectors by a backbone network, such as Resnet (He et al., 2016). Second, the last convolution layer feeds into a pose estimation sub-network and generates the position of body joints, which is used as the input of the action recognition sub-network. Third, the features of each joint are extracted by RoIAlign (He et al., 2017) according to the positions of body joints. After that, a non-linear transformation is performed to get an L dimensional feature vector for each body joint. Fourth, the features of body joints will be split into two branches: one branch is a joint spatial reasoning (JSR) sub-network, which is used to build a relation graph to capture position relations between joints. The other branch is an intra-joint temporal relation (IJTR) sub-network, which models the temporal information between body joints. Then, the output features of JSR and IJTR are fused to form joints spatial-temporal reasoning feature for recognizing action from videos.

### Pose estimation

**Figure 3:**
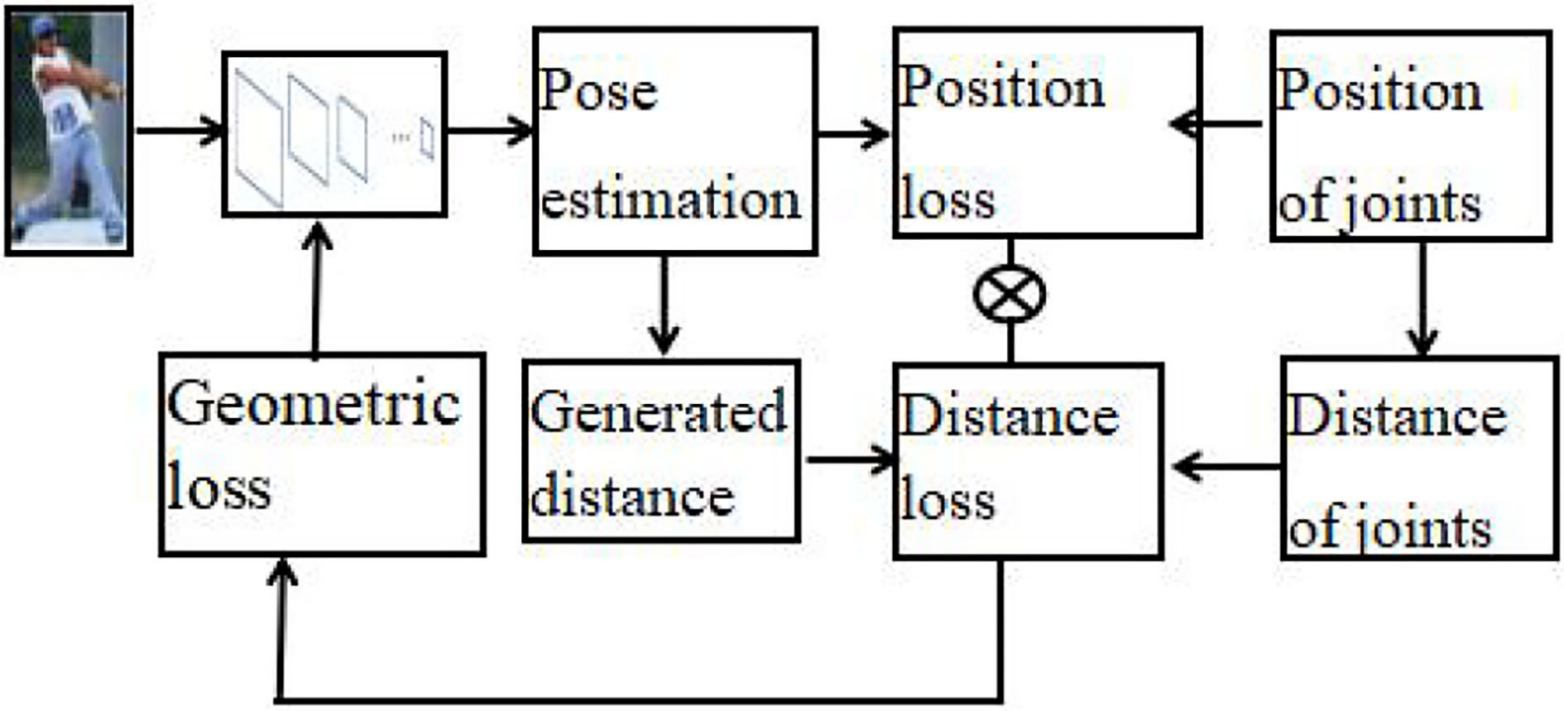
Geometric constraint-based human pose estimation.

The accuracy of pose estimation is important for our JSTR network, and it is a key module for feature extraction of body joints. To further improve the accuracy of pose estimation, we extend the structured feature learning framework (Xiao et al., 2016) and give a geometric constraint-based human pose estimation method (GCHP), which is illustrated in Figure 3.

We first apply the pose estimation CNN network to generate the positions of body joints and the distance between body joints can be obtained by the positions of body joints. Thus, the loss function of joints' distance can be obtained by calculating the real distance and the generated joint position. Let *d*_*ij*_ denotes the relative distance between the i-th joint and the j-th joint, and the loss function of joint distance can be expressed as follows.


(1) $$\begin{array}{l} L_{d}=\overset{N}{\underset{n=1}{\sum }}\overset{P}{\underset{i=1}{\sum }}d_{ij} \end{array}$$

where *L*_*d*_ is the loss function, N is the number of training images, and P is the number of body joints.

Let L_p_ is the loss function of joints' position, and it can be calculated by


(2) $$\begin{array}{l} L_{p}=\sum_{x}\sum_{y}m(x,y)\sum_{k}t_{x}(x,y)log(\frac{e^{z_{k}(x,y)}}{\underset{k^{\prime }}{\sum }e^{z_{k^{\prime }}(x,y)}}) \end{array}$$

where (x,y) is the position of the body joint, k is the index of the joints, t_k_(x,y) is the real label of the position (x,y), and z_k_(x,y) is the predicted value.

Geometric constraint function L is a combination of the position loss function and distance loss function, and it can be calculated by


(3) $$\begin{array}{l} L=L_{p}+\lambda L_{d} \end{array}$$

where λ is used to balance the weight of position loss function and distance loss function.

Note that our geometric constraints-based human pose estimation method can be directly inserted into the existing pose estimation pipeline. They can effectively strengthen feature representations and do not increase too many parameters.

### Action recognition framework

In the video action recognition sub-network, a joint spatial-temporal reasoning graph is built to perform relational reasoning on joints for action recognition. The positions of body joints are obtained by the pose estimation sub-network, which is the input of the last convolution layer of the video action recognition sub-network. Features of each joint are obtained by RoIAlign (He et al., 2017). Afterward, upon these original features and positions of joints, we build a spatial-temporal reasoning graph, where each node denotes a joint. For the spatial graph, each edge is a scalar weight, which is computed according to the two joints' features and their relative position. For the temporal graph, each edge represents the temporal information of the same joint. The GCN is applied to conduct relational reasoning based on a spatial-temporal reasoning graph. After graph convolution, the spatial-temporal relational representation for joints is generated. Then the spatial features and temporal features are fused to recognize the action from videos.

#### Building JSR graphs

Graph model has been found to be effective at representing the spatial and temporal relations in visual content. Therefore, the graph structure is utilized to model body joints relation for action recognition.

##### Graph definition

We consider a joints spatial relation (JSR) graph. The nodes V = {(*f*_*i*_, *p*_*i*_)|*i* = 1, 2, ..., *P*} are a set of P body joints, where *f*_*i*_ is the d-dimension appearance feature of the i-th joint, and *p*_*i*_ = (*x*_*i*_, *y*_*i*_) is coordinates of the i-th body joint. Let *A*∈*R*^*P* × *P*^ be the adjacent matrix of the JSR graph, which represents pair-wise relation among joints. The relation value A_ij_ is the affective coefficient, which is used to measure the intensity of the features of nodes.

To obtain sufficient representational power to capture the underlying relation between two joints, both distance information and angel information need to be considered. Moreover, the distance relation and angel relation have different position attributes. Therefore, the relation value can be defined as a composite function below:


(4) $$\begin{array}{l} {\text{A}}_{\text{ij}}=h\left(f_{d}\left(p_{i},p_{j}\right),f_{a}\left(p_{i},p_{j}\right)\right) \end{array}$$

where *f*_*d*_(*p*_*i*_, *p*_*j*_) denotes the distance relationship between two joints, and the angel relation is calculated by *f*_*a*_(*p*_*i*_, *p*_*j*_). The function f() is a fusion function that fuses distance and angel relation.

Next, we will give the definition of distance relation, angel relation, and the fusion function.

Let *p*_*i*_ = (*x*_*i*_, *y*_*i*_)(*i* = 1, 2, ..., *P*) be the coordinates of the i-th joints, the distance relationship between the i-th joint and the j-th joint can be expressed as:


(5) $$\begin{array}{l} f_{d}(p_{i},p_{j})=||l_{j}-l_{{}_{i}}|{|}_{2} \end{array}$$

Let *f*_*a*_(*p*_*i*_, *p*_*j*_) be the angel relation between the i-th and j-th body joints, and it can be expressed as:


(6) $$\begin{array}{l} f_{a}\left(p_{i},p_{j}\right)=\text{arctan}\frac{y_{j}-y_{i}}{x_{j}-x_{i}} \end{array}$$

The function h fuses distance and angel relation. In our experiments, we adopt the following function to compute the value of *A*_*ij*_ (Jianchao and Limin, 2019).


(7) $$A_{ij}=\frac{f_{d}(p_{i},p_{j})\text{exp}(f_{a}(p_{i},p_{j}))}{\sum_{j=1}^{P}f_{d}(p_{i},p_{j})\text{exp}(f_{a}(p_{i},p_{j}))}$$

where we perform normalization on each joint using the Softmax function so that the sum of all the related values of one actor node i will be 1.

#### Building IJTR graphs

Temporal information of body joints is a crucial cue for activity recognition. We model the temporal information of body joints by the intra-joint temporal relation (IJTR) Graph. The IJTR graph is represented by an adjacent matrix *A*^*T*^∈*R*^*P* × *P*^. During training, we randomly sample a set of K = 2 frames from the entire video and build IJTR Graph. Therefore, for the adjacent matrix *A*^*T*^ of the IJTR Graph, we directly set $A_{ij}^{T}=\text{1}$, if the i-th joint and the j-th joint are in two sample frames, and $A_{ij}^{T}=\text{0}$, otherwise. At testing time, a sliding window approach is used, and the features from all windows are mean-pooled to form a global activity feature.

#### Reasoning and training on graphs

The convolutional neural network is originally used to extract visual features of images or videos based on 2D or 3D filters. In contrast, a graph convolutional neural network (GCN) can perform message propagation from nodes to its neighbor nodes. Thus, it is usually used to perform relational reasoning. In this paper, GCN is applied to reasoning relations of human body joints.

Given a graph with N nodes, the operation of one graph convolution layer can be expressed as follows.


(8) $$X_{{}_{\left(l+1\right)}}=\sigma ({\left(D\right)}^{-\frac{1}{2}}A{\left(D\right)}^{-\frac{1}{2}}X_{\left(l\right)}W^{\left(l\right)})$$

where D∈ℜ^P×*P*^ is the degree matrix of A, $X_{{}^{\left(l\right)}}^{}$ is the output of the (*l*−1)-th layer, W1 is the learned parameters, and σ(.) is a non-linear activation function like ReLU.

In particular, in our JSTR framework, the adjacent matrix of the JSR is defined in Section Building IJTR graphs and the adjacent matrix of the IJTR is defined in Section Reasoning and training on graphs. ${\text{X}}^{\text{(0)}}=\left[f\left(x_{1}\right),f\left(x_{2}\right),...,f\left(x_{P}\right)\right]$ is the initial feature matrix, where *f*(*x*_*i*_) is the feature vector of the i-th joint. The final output of the GCN is updated features of joints, *X*_(*l*)_, in the graphs, which can be aggregated into a frame-level vector. In this paper, the GCN output of JSR and IJTR is aggregated into spatial relation features and temporal relation features, respectively, and then these two features are aggregated into spatial-temporal relation features for recognizing action from videos.

## Experiments

### Dataset

#### UCF101

UCF101 (Soomro et al., 2012) has 13,320 web video clips and 101 action classes. The videos range from daily life activities to unusual sports and each video. The average accuracy of three training/testing splits is adopted in our experiments.

#### HMDB51

The HMDB51 dataset (Kuehne et al., 2011) has 6, 766 videos downed from movies and web videos, and each video is labeled with one of the 51 action categories. The average accuracy of three training/testing splits is adopted in our experiments. Videos are subject to different viewpoints, video quality, camera motions, and occlusions.

#### Kinetics

The kinetics dataset is a large dataset for human action recognition, containing over 240,000 video action clips (Carreira et al., 2017). It uses a training set of 236,763 videos and a testing set of 19,095 videos. There are 400 types of actions.

### Implementation details

An input video is uniformly partitioned into 16 segments, and one frame is randomly Sampled from each segment to obtain 16 frames for one video. The pose estimation sub-network generates 16 body joints, and the features of each joint are extracted by RoIAlign and get a 1,024-dimensional feature vector for each joint. Two JSR and IJTR are pre-trained on the training set separately. The network is trained in 150 epochs using the minibatch size of 32. The learning rate starts from 0.001 and multiplies to 0.1 every 30 epochs.

### The evaluation of GCHP

The GCHP is evaluated on the MPII (Andriluka et al., 2014) dataset. It contains 25,000 images with 40,000 different human instances and uses a training set of about 28,000 human instances and a testing set of 11,000 human instances, and the total number of body joints used is 16. The PCP (percentage of correct parts) is employed in this experiment. Similar to structured feature learning, the strict PCP, which only when both ends lie within 50% of the ground truth will this prediction is correct, is used. We compare experimental results with three deep learning-based methods (Tang et al., 2018; Xiao et al., 2018; Yang et al., 2020; Zhang et al., 2021). Table 1 shows the experimental results on the MPII dataset. As we can see from Table 1, our GCHP outperforms previous state-of-the-art work on every body part evaluated.

**Table 1:** Experimental results on the MPII dataset.

**Experiment**	**Head**	**Shoulder**	**Elbow**	**Wrist**	**Hip**	**Knee**	**Ankle**	**Mean**
Tang et al. (2018)	95.6	95.9	90.7	86.5	89.9	86.6	82.5	89.8
Xiao et al. (2018)	97.0	95.9	90.3	85.0	89.2	85.3	81.3	89.6
Yang et al. (2020)	97.1	95.9	90.3	86.4	89.1	87.1	83.3	89.9
Zhang et al. (2021)	97.5	96.0	90.7	87.3	89.5	87.7	83.4	90.3
GCHP	98.1	96.2	90.8	87.4	89.9	86.7	83.6	90.6

### Ablation studies

In this subsection, we perform detailed studies on the action recognition datasets to understand the contributions of our model components to video action recognition. In our experiments, we selected 14 body joints for one frame and sampled 20 frames for one video.

#### Joints spatial reasoning

We first train the pose estimation sub-network on the MPII dataset (Andriluka et al., 2014), and the trained network is used to predict the position of body joints. In our experiments, we select 14 body joints including head, upper arm, lower arm, upper leg, lower leg, and etc. Then, ROIAlign is used to extract features of these body joints. JSR is built and Table 1 gives the results of the fusion of JSR and baseline method TSN (Wang et al., 2016; Zhang et al., 2021). We can observe that JSR can improve the accuracy by 0.7, 0.6, and 1.1%, respectively, on Kinetics, UCF101, and HMDB51 dataset.

#### Intra-joint temporal relation

We further evaluate the effect of IJTR. In the training phase, two adjacent video frames are selected to build the IJTR graph. In the test phase, we use a sliding window approach, and the emotion features from all windows are fused in a mean-pooled manner. Table 2 gives the results of the fusion of IJTR and baseline method TSN. As can be shown in Table 3, the accuracy improved by 0.3, 1.1, and 2.2%, respectively, on Kinetics, UCF101, and HMDB51 datasets.

**Table 2:** Exploration of SA.

**Method**	**Kinetics accuracy (%)**	**UCF101 accuracy (%)**	**HMDB51 accuracy (%)**
TSN	92.4	94.2	69.4
JSR	93.1	94.8	70.5

**Table 3:** Exploration of PR.

**Method**	**Kinetics accuracy (%)**	**UCF101 accuracy (%)**	**HMDB51 accuracy (%)**
TSN	92.4	94.2	69.4
IJTR	92.7	95.3	71.6

#### Joints spatial-temporal reasoning

The output of JSR and IJTR is fused and produce the feature of joints spatial-temporal reasoning. Table 4 gives the results of JSTR. Table 4 shows the comparison results of JSTR and baseline method TSN, we can see that the accuracy improved by 1.2, 2.1, and 3.0%, respectively, on kinetics, UCF101, and HMDB51 datasets.

**Table 4:** Exploration of JSTR.

**Method**	**Kinetics accuracy (%)**	**UCF101 accuracy (%)**	**HMDB51 accuracy (%)**
TSN	92.4	94.2	69.4
JSTR	93.6	96.3	72.4

### Comparison with the state-of-the-art

Now, we compare our model with the state-of-the-art methods. We compare our model with the state of the art methods on UCF101 and HMDB dataset. Our approach obtains robust improvements over the state-of-the-art methods. As can be seen from Table 5, the accuracy of our method on UCF101 and HMDB datasets is 96.3 and 72.4%, respectively. Compared with the state-of-the-art method, it increased by 1.5 and 0.2%, respectively, on the UCF101 and HMDB datasets. We also notice that the methods (Simonyan and Zisserman, 2014; Feichtenhofer et al., 2016a,b; Zhang et al., 2021) fuse spatial and temporal features without attention mechanism and relation reasoning, so their accuracy is relatively low. Attention cluster (Xiang et al., 2018) improves performance by first analyzing the importance of each local feature and then bestowing the global feature. Relation reasoning is adopted in MUSLE (Li D. et al., 2019) and the accuracy can be improved.

**Table 5:** Mean classification accuracy (%) compared with state-of-the-art methods on UCF101 and HMDB51.

**Method**	**UCF101 (%)**	**HMDB51 (%)**
Two Stream (Simonyan and Zisserman, 2014)	88	59.4
TSN (Zhang et al., 2021)	94	68.5
Fusion (Feichtenhofer et al., 2016a)	92.5	65.4
ST-ResNet (Feichtenhofer et al., 2016b)	93.4	66.4
Attention cluster (Xiang et al., 2018)	94.6	69.2
MUSLE (Li D. et al., 2019)	94.8	72.2
Our methods	96.3	72.4

On Kinetics, Table 6 shows the kinetics top-1 and top-5 accuracy on different methods. TSM (Lin et al., 2019) balances accuracy and computation cost by being inserted into 2D CNNs to achieve temporal modeling. The computation cost is relatively low, and the accuracy is also relatively low. STM (Jiang et al., 2019) uses a unified 2D CNN network that integrates spatial-temporal and motion features. It can extract long-term temporal features without 3D CNN, and the accuracy is higher than TSM. TEA (Li et al., 2020) was designed to capture both short- and long-range temporal evolution, which further improves the accuracy. MUSLE (Li D. et al., 2019) exploits the discriminative sub-graphs across different scales for facilitating spatio-temporal reasoning. The accuracy is higher than those methods without a reasoning relationship. As shown in Table 6, the top-1 and top-5 accuracy of our method are 75.8% and 93.6, respectively. It increases top-5 by 1.6% compared with the state-of-the-art method. There are two reasons that JSTR can achieve excellent performance. First, JSR can capture position relations between joints. Second, the temporal information of body joints is modeled.

**Table 6:** Kinetics top-1 and top-5 accuracy (%).

**Method**	**Top-1 (%)**	**Top-5 (%)**
TSM (Lin et al., 2019)	72.5	90.7
STM (Jiang et al., 2019)	73.7	91.6
TEA (Li et al., 2020)	74.0	91.3
MUSLE (Li D. et al., 2019)	75.1	92
Our methods	75.8	93.6

## Conclusion

To model the relation between body joints, a joint spatial-temporal reasoning (JSTR) framework is proposed. The JSTR consists of two sub-networks. In the pose estimation sub-network, geometric constraints-based human pose estimation is applied to further improve pose estimation accuracy. In the action recognition sub-network, a joints spatial reasoning (JSR) graph and intra-joint temporal relation (IJTR) graph are built, respectively, which is used to capture position and temporal relations between joints. Finally, the spatial reasoning feature and temporal reasoning feature are fused to recognize the action from videos. We validate JSTR in action recognition using three datasets, UCF101, HMDB51, and Kinetics. The experiment results show that JSTR can improve the recognition accuracy compared to the state-of-the-art methods. In terms of future work, we hope to extract spatio-temporal GCN features simultaneously to further improve the accuracy of action recognition and integrate it into end-to-end trained networks.

## Data availability statement

Publicly available datasets were analyzed in this study. This data can be found here: University of Central Florida (UCF) Center for Research in Computer Vision, https://www.crcv.ucf.edu/data/UCF101.php.

## Author contributions

All authors listed have made a substantial, direct, and intellectual contribution to the work and approved it for publication.

## Funding

This work was supported by the foundation of the He'nan Educational Committee (21A520006) and the scientific and technological research project of the Henan Provincial Science and Technology Department (182102310919).

## Conflict of interest

The authors declare that the research was conducted in the absence of any commercial or financial relationships that could be construed as a potential conflict of interest.

## Publisher's note

All claims expressed in this article are solely those of the authors and do not necessarily represent those of their affiliated organizations, or those of the publisher, the editors and the reviewers. Any product that may be evaluated in this article, or claim that may be made by its manufacturer, is not guaranteed or endorsed by the publisher.

## References

[B1] Andriluka M. Pishchulin L. Gehler P. Schiele B. (2014). “Human pose estimation: new benchmark and state of the art analysis,” in IEEE International Conference on Computer Vision and Pattern Recognition (Columbus, OH: IEEE), 3686–3693. 10.1109/CVPR.2014.471

[B2] Carreira J. Zisserman A. Vadis Q. (2017). “Action recognition? A new model, and the kinetics dataset,” in IEEE International Conference on Computer, Vision, and Pattern, Recognition (Honolulu, HI: IEEE), 4724–4733. 10.1109/CVPR.2017.502

[B3] Chéron G. Laptev I. Schmid C. (2015). “P-cnn: pose-based cnn features for action recognition,” in Proceedings of the IEEE International Conference on Computer Vision (Santiago: IEEE), 3218–3226. 10.1109/ICCV.2015.368

[B4] de Carvalho K. B. Basílio V. T. Brandão A. S. (2022). Action recognition for educational proposals applying concepts of Social Assistive Robotics. Cogn. Syst. Res. 71, 1–8. 10.1016/j.cogsys.2021.09.002

[B5] Defferrard M. Bresson X. Van dergheynst P. (2016). “Convolutional neural networks on graphs with fast localized spectral fifiltering,” in Advances in Neural Information Processing Systems (Barcelona: MIT Press), 3844–3852.

[B6] Fang S. Liu F. Xiong Z. Fang J. Li L. (2021). Seismic performance evaluation of recycled aggregate concrete-filled steel tubular columns with field strain detected via a novel mark-free vision method. Structures 37, 426–441. 10.1016/j.istruc.2021.12.055

[B7] Feichtenhofer C. Pinz A. Wildes R. P. (2016b). “Spatiotemporal residual networks for video action recognition,” in Advances in Neural Information Processing Systems (Honolulu, HI: IEEE), 3468–3476. 10.1109/CVPR.2017.787

[B8] Feichtenhofer C. Pinz A. Zisserman A. (2016a). “Convolutional two-stream network fusion for video action recognition,” in IEEE International Conference on Computer Vision and Pattern Recognition (Las Vegas, NV: IEEE), 1933–1941. 10.1109/CVPR.2016.213

[B9] He K. Gkioxari G. Dollar P. Girshick R. B. (2017). “Mask R-CNN,” in IEEE International Conference on Computer Vision (Venice: IEEE), 2980–2988.

[B10] He K. Zhang X. Ren S. J. J. Sun. (2016). “Deep residual learning for image recognition,” in IEEE International Conference on Computer Vision and Pattern Recognition (Las Vegas, NV: IEEE), 770–778. 10.1109/CVPR.2016.90

[B11] Jianchao W. Limin W. (2019). “Learning actor relation graphs for group activity recognition,” in IEEE International Conference on Computer Vision and Pattern Recognition (Seoul: IEEE), 9964–9974. 10.1109/CVPR.2019.01020

[B12] Jiang B. Wang M. Gan W. Wu W. Yan J. (2019). “STM: spatiotemporal and motion encoding for action recognition,” in IEEE International Conference on Computer Vision (Seoul: IEEE), 2000–2009. 10.1109/ICCV.2019.00209

[B13] Kuehne H. Jhuang H. Garrote E. Poggio T. Serre T. (2011). “Hmdb: a large video database for human motion recognition,” in IEEE International Conference on Computer Vision (Barcelona: IEEE), 2556–2563. 10.1109/ICCV.2011.6126543

[B14] Li D. Qiu Z. Pan Y. Yao T. Li H. Mei T. (2019). “Representing videos as discriminative sub-graphs for action recognition,” in International Conference on Computer Vision and Pattern Recognition (Long Beach, CA: IEEE), 3310–3319.

[B15] Li M. Chen S. Chen X. Zhang Y. Wang Y. Tian Q. (2019). “Actional-structural graph convolutional networksfor skeleton-based action recognition,” in International Conference on Computer Vision and Pattern Recognition (Long Beach, CA: IEEE), 3595–3603.

[B16] Li Y. Ji B. Shi X. Zhang J. Kang B. Wang L. (2020). “TEA: temporal excitation and aggregation for action recognition,” in International Conference on Computer Vision and Pattern Recognition (Seattle, WA: IEEE), 906–915.

[B17] Li Z. Gavves E. Jain M. Snoek C. G. M. (2018). VideoLSTM convolves, attends and flows for action recognition. Comput. Vision Image Understand. 166, 41–50. 10.1016/j.cviu.2017.10.011

[B18] Lin J. Gan C. Han S. (2019). “TSM: temporal shift module for efficient video understanding,” in International Conference on Computer Vision (Seoul: IEEE), 7082–7092. 10.1109/ICCV.2019.00718

[B19] Simonyan K. Zisserman A. (2014). “Two-stream convolutional networks for action recognition in videos,” in Advances in Neural Information Processing Systems (Montreal, QC: MIT Press), 568–576.

[B20] Soomro K. Zamir A. R. Shah M. (2012). UCF101: A Dataset of 101 Human Actions Classes From Videos in The Wild. Available online at: https://ui.adsabs.harvard.edu/abs/2012arXiv1212.0402S/abstract

[B21] Sun L. Jia K. Yeung D. Y. Shi B. E. (2015). “Human action recognition using factorized spatio-temporal convolutional networks,” in IEEE International Conference on Computer Vision (Santiago: IEEE), 4597–4605. 10.1109/ICCV.2015.522

[B22] Tang W. Yu P. Wu Y. (2018). “Deeply learned compositional models for human pose estimation,” in Proceedings of the European Conference on Computer Vision (Cham: Springer), 197–214. 10.1007/978-3-030-01219-9_12

[B23] Tang Y. Chen M. Wang C. Luo L. J. Lian G. Zou X. (2020). Recognition and localization methods for vision-based fruit picking robots: a review. Front. Plant Sci. 11, 510. 10.3389/fpls.2020.00510 32508853 PMC7250149

[B24] Tran D. Bourdev L. Fergus R. Torresani L. Paluri M. (2015). “Learning spatiotemporal features with 3d convolutional networks,” in Proceedings of the IEEE International Conference on Computer Vision (Santiago: IEEE), 4489–4497. 10.1109/ICCV.2015.510

[B25] Wang L. Xiong Y. Wang Z. Qiao Y. Lin D. Tang X.. (2016). “Temporal segment networks: towards good practices for deep action recognition,” in European Conference on Computer Vision (Cham: Springer), 20–36. 10.1007/978-3-319-46484-8_2

[B26] Wang Y. Long M. Wang J. Yu P. S. (2017). “Spatiotemporal pyramid network for video action recognition,” in IEEE International Conference on Computer Vision and Pattern Recognition (Honolulu, HI: IEEE), 2097–2106. 10.1109/CVPR.2017.226

[B27] Wu F. Duan J. Ai P. Chen Z. Yang Z. Zou X. (2022). Rachis detection and three-dimensional localization of cut off point for vision-based banana robot. Comput. Electron. Agric. 198, 107079. 10.1016/j.compag.2022.107079

[B28] Xiang L. Gan C. Melo G. D. Wu J. Xiao L. (2018). “Attention clusters: purely attention based local feature integration for video classification,” in IEEE International Conference on Computer Vision and Pattern Recognition (Salt Lake City, UT: IEEE), 7834–7843.

[B29] Xiao B. Wu H. Wei Y. (2018). “Simple baselines for human pose estimation and tracking,” in Proceedings of the European Conference on Computer Vision (Cham: Springer), 472–487. 10.1007/978-3-030-01231-1_29

[B30] Xiao C. Ouyang W. Li H. Wang X. (2016). “Structured feature learning for pose estimation,” in IEEE International Conference on Computer Vision and Pattern Recognition (Las Vegas, NV: IEEE), 4715–4723.

[B31] Yan S. Xiong Y. Lin D. (2018). “Spatial temporal graph convolutional networks for skeleton-based action recognition,” in AAAI Conference on Artifificial Intelligence (New Orleans, LA: AAAI), 7444–7452. 10.1609/aaai.v32i1.12328

[B32] Yang C. An Z. Zhu H. Hu X. Xu Y. (2020). Gated convolutional networks with hybrid connectivity for image classification. Proc. AAAI Conf. Artif. Intell. 34, 12581–12588. 10.1609/aaai.v34i07.6948

[B33] Zhang C. He N. Sun Q. Yin X. Lu K. (2021). “Human pose estimation based on attention multi-resolution network,” in International Conference on Multimedia Retrieval (Taipei: ACM), 682–687. 10.1145/3460426.3463668

